# Current economic and regulatory challenges in developing antibiotics for Gram-negative bacteria

**DOI:** 10.1038/s44259-025-00123-1

**Published:** 2025-06-11

**Authors:** Nupur Gargate, Mark Laws, Khondaker Miraz Rahman

**Affiliations:** School of Cancer and Pharmaceutical Sciences, King’s College London, London, SE1 9NH UK

**Keywords:** Medicinal chemistry, Drug discovery, Health care, Medical research, Antimicrobials, Antibiotics, Antimicrobial resistance

## Abstract

Antimicrobial resistance (AMR) is a serious global threat projected to cause 10 million deaths annually by 2050. Antibiotics are becoming ineffective, leading to poor health outcomes and economic burden. Despite the urgent need, scientific, economic, and regulatory challenges hinder antibiotic development, causing major companies to exit the field. This review explores the AMR crisis, challenges in antibiotic development, particularly for Gram-negative bacteria, and potential solutions to revitalise the antibiotic pipeline.

## Antimicrobial resistance

Antimicrobial resistance (AMR) refers to the ability of microorganisms like bacteria, fungi, and viruses to resist antimicrobial action over time, leading to the emergence of drug-resistant pathogens. These pathogens counteract antimicrobials, rendering them ineffective and facilitating microbial spread^1,2^. The failure of antibiotics to act against these microbes has serious implications since the treatment of infections becomes complicated. The fact that bacteria have developed resistance to almost all classes of antibiotics has led to uncertainty for the future of bacterial infection treatment. Considering the prevalence and rise of AMR in the 21st century, the World Health Organisation (WHO) and Centers for Disease Control and Prevention (CDC) have declared it a global public health problem. Being one of the major concerns underpinning healthcare today, the AMR situation requires immediate action.

**Fig. 1:**
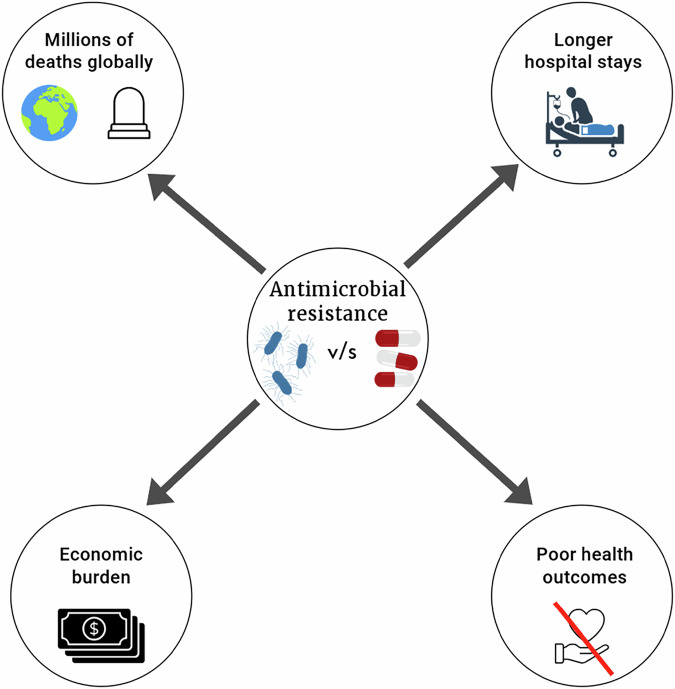
The global impact of antimicrobial resistance. The figure illustrates the significant global impact of antimicrobial resistance. AMR leads to severe consequences, including a rising number of deaths worldwide. It also contributes to longer hospital stays, which highlights the challenges in managing resistant infections. The economic burden of AMR is evident through increased healthcare costs and productivity losses. Additionally, it results in poor health outcomes, which affect the quality of life and recovery prospects. The interconnected arrows emphasise how AMR’s multifaceted effects ripple through healthcare, economies, and global health.

As of 2021, 4.71 million deaths globally were estimated to be associated with AMR. Previous estimates projected that, without effective measures, AMR could lead to 10 million deaths annually by 2050^3^. Despite global efforts over the past decade, the burden remains high, with projections indicating 8.22 million deaths associated with AMR and 1.91 million deaths directly attributable to it by 2050. Geographically, the highest all-age mortality rates are expected to occur in South Asian, Latin American, and Caribbean countries^4^. Along with its impact on patient lives, AMR significantly increases the burden on healthcare systems, since patients with resistant infections have limited treatment options and require longer hospital stays, more isolation, and preventive measures to restrict nosocomial spread (Fig. 1). Surgical procedures and chemotherapy too become complicated, since these procedures are heavily antibiotic-dependent. Treating patients with such resistant infections could add up to US$29,000 per patient in hospitals^5^. Furthermore, AMR would also burden the global economy, as studies have reported it to cost US$1 trillion per year^6^.

The reasons for AMR are multifactorial; however, the overuse of antimicrobials, inappropriate prescribing of antibiotics, and their misuse for incorrect treatment indications are considered major contributing factors^2^. Over-the-counter sales of antibiotics in low and middle-income countries (LMICs) enhance their accessibility and consumption even when unneeded. A study on antibiotic accessibility and use in LMICs found that Bangladesh and Vietnam had the highest proportion of unlicensed antibiotic selling shops, with self-medication being widespread due to easy accessibility of antibiotics among the general public^7^. In Vietnam specifically, 97% of patients were prescribed antibiotics for acute respiratory infections, as per a case study. Among these prescriptions, 92.5% were Access antibiotics- classified by the WHO for treatment of common infections- while 5.6% were Watch antibiotics, which have a higher potential for resistance development^8,9^. Alarmingly, in India, China, and Kenya, approximately 30%–50% of antibiotic prescriptions are issued for conditions such as asthma, angina, and acute diarrhoea—cases in which antibiotic therapy is both irrational and clinically unwarranted. This high rate of misuse is a serious concern as it accelerates resistance without improving outcomes, contributing further to the global AMR burden^10^. The usage of these life-saving drugs in LMICs has also been shown to increase with increasing income, frequent hospitalisation, and high prevalence of hospital infections^11^.

On the other hand, in high-income countries, greater antibiotic use in clinical settings and commercial use contributes to overuse^2,11^. Agricultural use of antibiotics in animals is also a significant contributor to resistance. About 73% of the antibiotics used in humans are also used in agriculture^12^. Using them to promote growth and treat and prevent animal infections results in resistance, which is then transferred to humans through food consumption and contact^13^. While many high-income countries have restricted or banned the use of antibiotics for growth promotion in animals, there are a few exceptions where such bans are not fully implemented or where loopholes exist. For example, in high-income countries such as the USA, Canada, and Australia, official bans on growth promotion are in place; however, preventive use remains widespread, potentially undermining the intent of these regulations. This contrasts with the European Union, where both growth promotion and routine prophylactic use of medically important antibiotics are largely banned under stricter controls.

## The dwindling antibiotic pipeline

The development of antibiotics was the turning point in human history, significantly reducing mortality from bacterial infections and improving life expectancy. Salvarsan, penicillin, and prontosil marked the beginning of the antibiotic era in the early 1900s due to their success in treating syphilis and streptococcal infections. Between the 1940s and 1960s, antibiotic discovery reached its golden era, witnessing the development of more than 20 new antibiotic classes^14^. This rapid progress was driven by the Waksman platform, developed by microbiologist Selman Waksman, which exploited the natural ability of bacteria to produce antibiotics as a means of competing with other microorganisms in their environment. This approach involved isolating and testing actinomycetes, leading to the discovery of streptomycin and numerous other antibiotics through the 1970s. However, due to the constant exploitation of soil bacteria over decades, the platform eventually collapsed and failed to yield significant results in later years^15^.

**Fig. 2:**
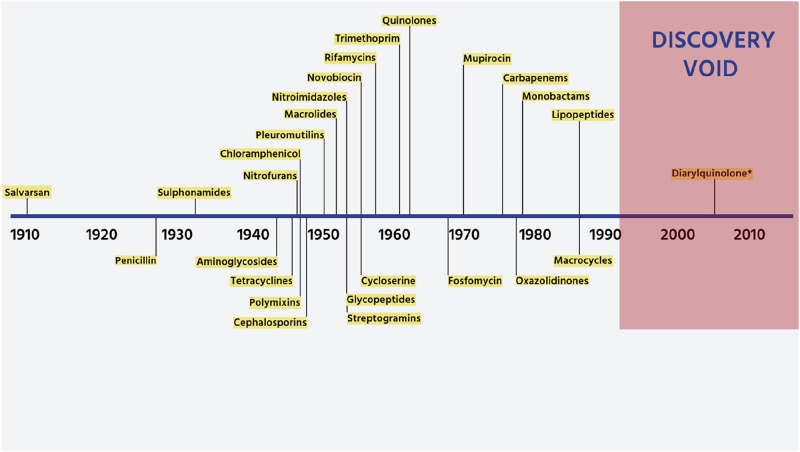
The antibiotic discovery void. The figure shows the timeline of antibiotic discovery from 1910 to the 2010s, highlighting the golden era of innovation (1940–1960) when major antibiotic classes, including penicillin, aminoglycosides, tetracyclines, and macrolides were discovered and developed. After 1960, the discovery rate sharply declined, leading to the “discovery void”, shown in pink, spanning the late 20th and early 21st centuries. This void reflects a stagnation in the discovery of new antibiotic classes, with only diarylquinolone introduced during this period. The timeline highlights the urgent need for new antibiotics to combat antimicrobial resistance, as innovation has failed to keep pace with evolving bacterial resistance.

As a result, following the 1980s, the discovery of new antibiotics started diminishing. The antibiotic analogues failed to keep pace with the emergence of resistance, and only 5 novel classes of antibiotics have been marketed since 2000- oxazolidinones, lipopeptides, pleuromutilins, tiacumicins, and diarylquinolines^16^. The period following 1987 is hence referred to as the “antibiotic discovery void”^17,18^ (Fig. 2).

The depletion of the antibiotic pipeline was further exacerbated by the exit of several major pharmaceutical companies from antibacterial R&D. Since the 1990s, 18 major pharmaceutical companies have reportedly exited the field. Although four big pharmaceutical companies- GSK, Novartis, Sanofi, and AstraZeneca- maintained active antibacterial programmes beyond this period, they too have shifted from the field between 2016 and 2019^16,19^. The declining interest in antibiotic R&D among pharmaceutical companies can be attributed to a combination of scientific, regulatory, and financial challenges. While bacterial resistance and reduced antibiotic susceptibility are significant scientific challenges, it is mainly the economic barriers that deter pharmaceutical companies from investing in this field^20,21^. Due to the short duration of antibiotic treatments, sales and returns on investment are considerably lower compared to other classes of medication. Consequently, despite substantial investment in antibiotic discovery and development, pharmaceutical companies struggle to recoup development costs and often choose to allocate their resources to fields such as cardiovascular medicine and oncology, which generate sustained revenue due to longer treatment durations and premium pricing^22^. This, along with the lack of expertise in antibiotic R&D, meagre funding for antibiotic research, and limited business models, are contributing to the dwindling antibiotic pipeline.

A detailed review of candidates currently in the antibacterial pipeline has been published by the WHO in June 2024 to drive efforts in addressing critical unmet medical needs^23^. First released in 2017, this report represents the fourth review of preclinical antibacterial agents and the sixth annual review of those in clinical development. It also references the WHO’s Bacterial Priority Pathogen List (BPPL), a strategic framework to help direct research funding and innovation efforts. As of the 2023 analysis, the current antibacterial pipeline includes 97 antibacterial agents, including 57 traditional antibiotics and 40 non-traditional therapies. Of the traditional agents, 32 target the pathogens listed in the BPPL, but only 12 meet at least one of the WHO’s innovation criteria (i.e., no cross-resistance, new target, new mode of action, and/or new class), highlighting the limited novelty in the pipeline. Moreover, only four of these innovative candidates target at least one *critical* pathogen from the WHO BPPL, further emphasising the urgent need for the new therapies against priority pathogens. With respect to antibiotics active against Gram-negative bacteria, increased efforts since 2017 have been observed. 50 antimicrobial agents are in various phases of clinical trials and target Enterobacterales, *Pseudomonas aeruginosa*, and *Acinetobacter baumannii*, including 28 traditional and 21 non-traditional candidates^24^. Overall, while there has been a rising trend in the number of antibacterial candidates in development, the report states that these agents are still insufficient to tackle the increasing spread of drug-resistant infections^25^.

Furthermore, the pipeline is dominated by multiple analogues of the existing classes, particularly β-lactamase inhibitor combinations. Only two agents approved since 2017- vaborbactam and lefamulin- represent a new chemical class, while more than 80% belong to existing classes. This trend arises because developing new analogues by modifying existing classes carries lower risk and requires less investment in R&D. However, while this approach may buy time in the fight against AMR, it is not a long-term solution to reinvigorating the antibiotic pipeline. Most antibiotic classes face multiple resistance mechanisms, and simple chemical modifications cannot overcome all of them, leaving some degree of cross-resistance with existing classes^14,26,27^. Therefore, the development of novel antibacterial agents with a different mechanism of action or targets is essential, but this critical need remains largely unaddressed by the current pipeline.

Considering the threat and implications of this silent pandemic, the United Nations General Assembly (UNGA) convened its first High-level meeting (HLM) on AMR in 2016. This meeting acknowledged the multifaceted nature of AMR and endorsed a One Health Approach, addressing human, animal, and environmental health as interconnected components in tackling the crisis. Building on this foundation, the second UNGA HLM on AMR was held in September 2024 under the theme “investing in the present and securing our future together”^28^. In the meeting, the political leaders pledged to reduce AMR-associated deaths by 10% by 2030 and called for catalytic funding of US$100 million to help achieve the target of 60% of the countries globally having funds to execute AMR plans by 2030^29^.

## Prevalence of Gram-negative bacterial infections

In parallel with the 2023 antibacterial pipeline review, the WHO’s BPPL—originally published in 2017 and most recently revised in 2024—continues to highlight pathogens for which novel antibiotic development is urgently needed^30^. It categorises pathogens into *critical, high*, and *medium* priority based on several criteria, including disease burden; prevalence of resistance and mortality; issues related to preventability, treatability, and transmissibility; and the urgency of therapeutic innovation^31^. Among the pathogens designated as *critical* are members of the ESKAPE (i.e., *Enterococcus faecium, Staphylococcus aureus, Klebsiella pneumoniae, Acinetobacter baumannii, Pseudomonas aeruginosa*, and *Enterobacter* spp.) group. Notably, 3 pathogens in this group are Gram-negative- *Klebsiella pneumoniae*, *Acinetobacter baumannii*, and *Pseudomonas aeruginosa*. Their continued classification as *critical* in the 2024 revision underscores their persistent role in driving antimicrobial resistance and associated mortality^30^.

These three pathogens present a massive threat in hospital settings as they are virtually resistant to all antibiotic classes. Resistance to carbapenems, cephalosporins, beta-lactams, aminoglycosides, and fluoroquinolones has been reported for these bacteria, where some of the isolates are resistant to more than three classes of antibiotics at the same time (called multidrug-resistant species)^32^. The resistance against carbapenems is particularly concerning since this class is considered the last resort for treating serious Gram-negative bacterial infections^33^. While carbapenem-resistant *K. pneumoniae* continues to pose a significant threat, *A. baumannii* is also considered notorious since multidrug resistance rates for this pathogen have been observed to be 90% or more in Greece, Turkey, and Romania^34^.

Furthermore, numerous hospital-acquired infections (HAI), including hospital-acquired pneumonia, urinary tract infections, and bloodstream infections, along with device-associated infections like ventilator-associated pneumonia and catheter-associated urinary tract infections, have been encountered due to these opportunistic pathogens^35,36^. A study reported that *P. aeruginosa,* followed by *Klebsiella,* are among the most commonly encountered bacteria in Intensive Care Unit (ICU) settings^37^. Another study conducted over five years recorded that *E. coli* was the primary causative agent in HAI, followed by *Klebsiella* species. The study also emphasised that HAIs were four times more likely to be caused by Gram-negative pathogens than Gram-positive^38^. As of 2022, the mortality rates associated with Enterobacterales like *Klebsiella* have been reported to be up to 40%, while those for *A. baumannii* and *P. aeruginosa* are as high as 57% and 31%, respectively^39^.

Rising global resistance and mortality rates due to Gram-negative infections are concrete evidence that the situation is widespread and critical. The prevalence of infections and limited treatment options are strong indicators of a problematic future if action is not taken. The ideal solution in this scenario seems obvious – to develop new antibiotics that target these pathogens; the reality, however, is far from this simple, since antibiotic R&D is complex and associated with many challenges of different kinds. While scientific challenges in finding new molecules persist for almost all types of drugs, a host of unique economic and regulatory challenges also plague the antibiotic sector. This review will focus mainly on the latter.

## Challenges in developing antibiotics against Gram-negative bacteria

Bacteria can be classified as Gram-positive or Gram-negative, based on their cell wall structure differences. Due to the presence of an additional outer membrane that limits drug entry inside Gram-negative bacteria, developing antibiotics is significantly challenging. Along with the complex bacterial structure, low profitability and stringent regulatory requirements contribute to the difficulties in antibiotic R&D. The 3 major challenges facing antibiotic discovery and development can hence be classified as scientific, economic, and regulatory^40–44^.

## Scientific challenges

**Fig. 3:**
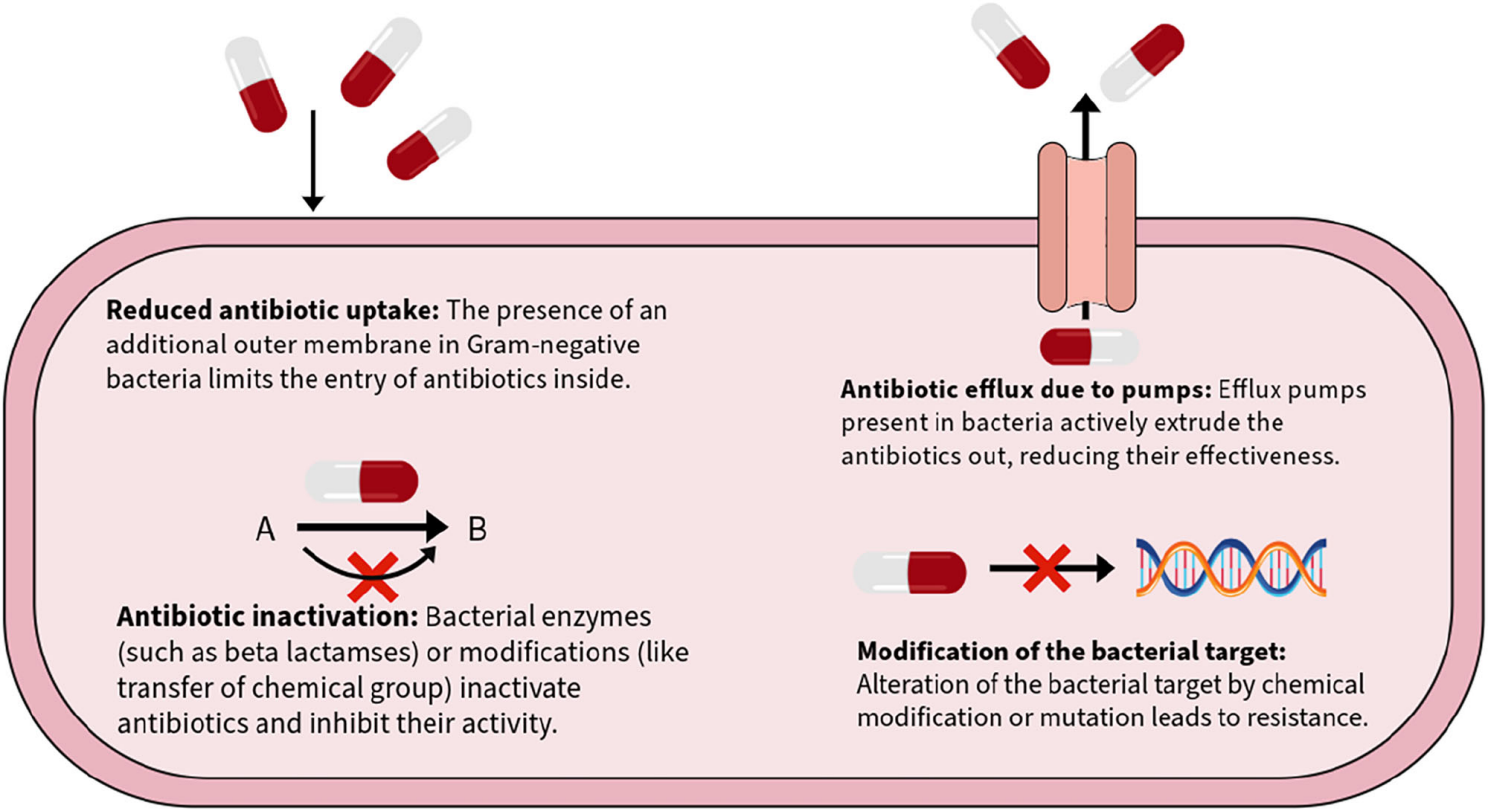
Major mechanisms contributing to antibiotic resistance. The figure illustrates key mechanisms of antibiotic resistance in bacteria. Reduced antibiotic uptake occurs when the outer membrane of Gram-negative bacteria prevents antibiotic entry. Antibiotic inactivation results from bacterial enzymes, such as beta-lactamases, or chemical modifications that deactivate antibiotics. Efflux pumps actively expel antibiotics, reducing intracellular drug concentration and effectiveness. Lastly, modification of bacterial targets, through mutations or chemical alterations, diminishes the antibiotic’s ability to bind and exert effects. These combined mechanisms highlight bacterial adaptations to survive antibiotic treatments, highlighting the complexity of bacterial resistance and the need for innovative strategies to combat antimicrobial resistance.

The scientific challenges of antibiotic development relate to bacterial structure and are primarily responsible for the emergence and spread of antimicrobial resistance. Bacteria employ four main resistance mechanisms: inactivation of the antibiotic, modification of drug target, limited permeability of Gram-negative outer membrane, and drug efflux (Fig. 3). Antibiotic inactivation occurs either through chemical modification of the antibiotic by enzymes- preventing target binding, or through enzymatic degradation of its structure- rendering it ineffective. Notable examples of enzymes that employ the latter mechanism include extended-spectrum beta-lactamases (ESBLs), which confer resistance to penicillins, cephalosporins, and monobactams, and carbapenemases, which target carbapenems. The production and carriage of these enzymes in Gram-negative bacteria is largely concerning since it limits the use of nearly the entire beta-lactam class, further complicating treatment options for life-threatening infections^45^. Target modification is another widespread mechanism, often resulting from point mutations in genes encoding bacterial enzymes such as DNA gyrase and topoisomerase IV (leading to fluoroquinolone resistance), or from enzymatic alterations like 16S rRNA methylation by *erm* genes, which block macrolide binding^45,46^.

A resistance mechanism unique to Gram-negative bacteria is low antibiotic permeability due to a structurally complex cell wall. The outer membrane in these bacteria acts as a significant barrier by limiting antibiotic influx and thus providing intrinsic resistance to several antibiotics that are effective against Gram-positive bacteria. Further modifications to the components of the outer membrane, such as porins or phospholipids, also alter antibiotic entry^47^. Complementing this permeability barrier, efflux pumps actively extrude antibiotics outside the bacteria in an energy-dependent manner, significantly contributing to resistance in Gram-negative bacilli. Among the six families of efflux pumps, the resistance-nodulation-cell division (RND) family is found in Gram-negative bacteria and is the most clinically relevant. A well-characterised example, AcrAB-TolC in *E. coli*, extrudes a range of antibiotics, including but not limited to tetracyclines, fluoroquinolones, chloramphenicol, and some beta-lactam antibiotics^46,48^. More thorough reviews of this area can be found elsewhere^45,46,49^.

## Economic challenges

A gradual turn of investors and pharmaceutical companies towards investment in non-communicable diseases like cancer began in the latter half of the 20th century. It was fuelled by the belief that infectious diseases are a problem of the past, ultimately leading to their divestment from the antibacterial portfolio. The current antibiotic crisis facing the world today stems partly, if not completely, from the economic challenges associated with antibiotic development, and the pivotal reason for this is the lack of profitability of these drugs. Antibacterials today capture less than 5% of the venture capital share, with only about US$1.8 billion invested in antibiotics out of a total of US$38 billion for pharmaceutical research and development^50^. Over the last 40 years, the number of antibiotics being approved by the US FDA has also dropped to 6% from 20% in 1980^51^.

**Fig. 4:**
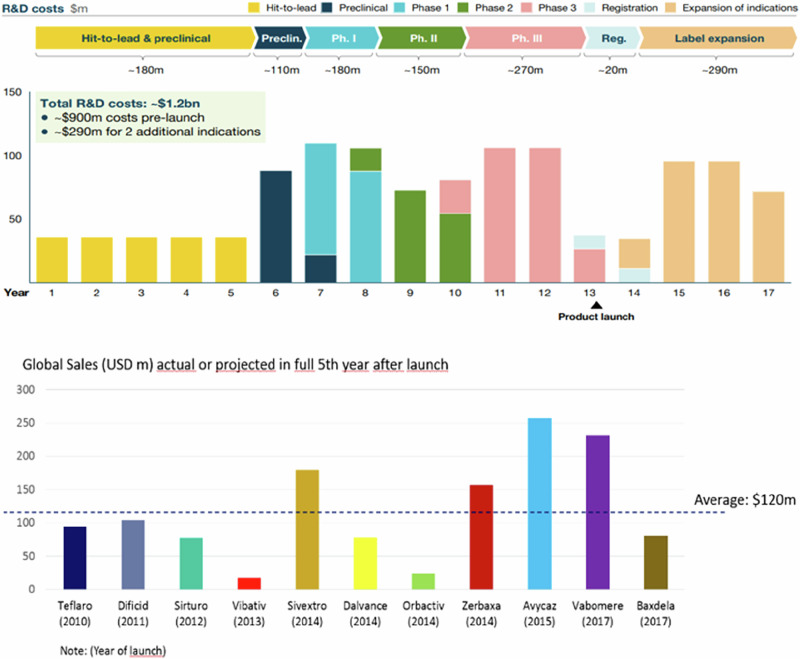
Comparison between the development cost of new antibiotics and their global sales^149,150^. (Top chart adapted from the Wellcome Trust with permission.) The figure compares antibiotic R&D costs and global sales. The top chart outlines R&D stages, spanning hit-to-lead, preclinical, and clinical trials (Phases I–III), with ~$1.2 billion total costs. Pre-launch expenses dominate at ~$900 million, with ~$290 million allocated for label expansions. Phase III is the costliest, reaching ~$270 million. The timeline highlights a ~13-year journey from discovery to launch. The bottom chart displays the fifth-year sales of recent antibiotics, with an average revenue of ~$120 million, significantly lower than R&D costs. Only a few drugs surpass the $100 million benchmark.

It is said that the antibiotics market is broken since the gap between the development cost of a new antibiotic and its sales is so enormous that no pharmaceutical company can afford to fix it. On average, the cost of developing antibiotics is around US$1.2 billion, yet stewardship practices often limit its use, resulting in sales about US$100 million or less, approximately a 10-fold lower value^52,53^ (Fig. 4). With such stark differences in investment and returns, it is incredibly difficult to make a profit; this is the major reason why the number of new antibiotics entering the market is low despite the urgent need for them.

**Fig. 5:**
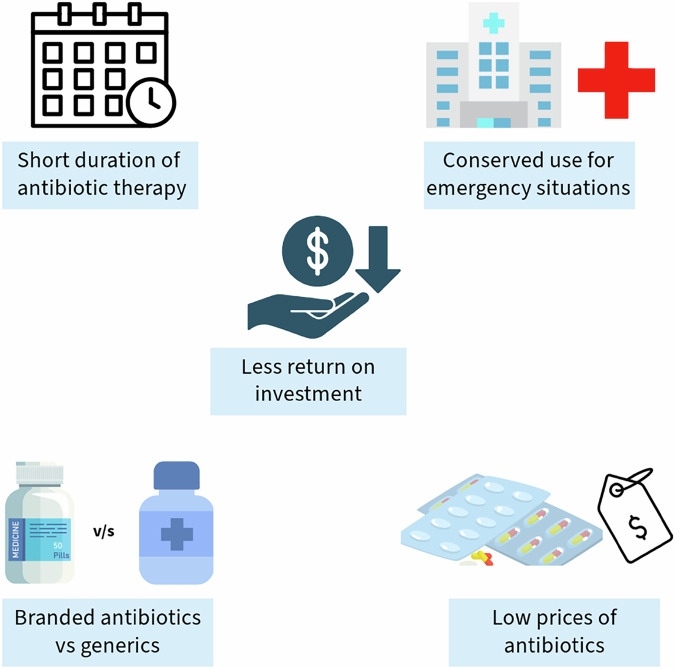
A summary of the economic challenges of antibiotic R&D. The figure outlines key economic challenges affecting antibiotic development and commercialisation, emphasising limited financial returns. Short treatment durations reduce sales volumes, making antibiotics less profitable than chronic therapies. The conservation of antibiotics for emergency use further limits their market demand, as these drugs are reserved to combat resistance. Additionally, the dominance of generic antibiotics over branded ones drives down revenue potential. Low pricing strategies for antibiotics, intended to ensure affordability, further exacerbate financial constraints for developers. Combined, these factors result in less return on investment, discouraging pharmaceutical companies from investing in antibiotic research and development, ultimately contributing to the current innovation gap in combating antimicrobial resistance.

But why is the business so bad for antibiotics in particular? The reasons are myriad (Fig. 5). Firstly, antibiotics are used for treating bacterial infections only briefly, for around 1–2 weeks. In contrast, diseases like cancer and diabetes require potential life-long usage of drugs for cure. This naturally lowers the sales and return on investment of antibacterials, making them unattractive for drug companies to invest in; revenue generated from cancer treatments is in billions, while that from an antibiotic’s sale is only US$46 million per year^54,55^. Pharmaceutical companies, hence, prefer to invest their money in other therapeutics with greater potential for profitability. The risk-adjusted net present value (rNPV) is used to predict the value of a pharmaceutical project by considering cash flows, capital investment, and the risks associated. It was reported that the rNPV value for antibiotics is only around US$100 million, as opposed to that for musculoskeletal drugs at US$1150 million, depicting the low financial viability of the antibiotics market^56^.

Secondly, the nature of antibiotics places them in a rather conflicting situation – on one hand is the necessity of conserving use to prevent resistance, and on the other is lower antibiotic sales as a direct consequence. Newly developed antibiotics are preserved to maintain their effectiveness, and while this ensures that resistance developed against them is limited, it greatly hinders their profitability, perhaps explaining why pharmaceutical companies choose to exit from antibacterial R&D^57,58^. No company would wish to spend billions of dollars, lots of resources, and decades of time only to discover and develop a new drug that is reserved for last resort use, and that too for only a short duration. Antibiotics thus face the “fire extinguisher problem”- inasmuch as they are necessary to hold in case of emergency, they have no value and cannot be used until a fire (in this case, bacterial resistance) occurs^19^. Moreover, since the phenomenon of resistance is inevitable, the need to develop new antibiotics shall always persist. Antibiotic R&D is ultimately stuck in a loop, where the development of new antibacterials drives resistance, and the spread of resistance compels new antibiotic discovery. Efficient diagnostics and infection prevention practices are hence required to ensure that in the fight against resistance, new molecule discovery does not take the burden alone.

**Fig. 6:**
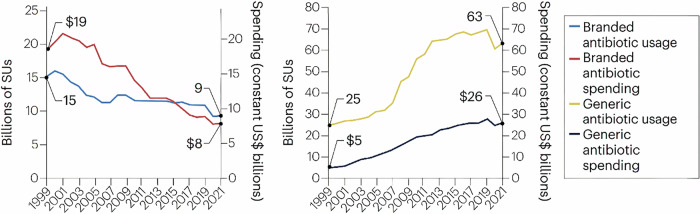
Comparison between the use of branded antibiotics and their generic counterparts (Adapted with permission from Madden and Outterson^61^). The figure compares antibiotic usage and spending for branded and generic drugs from 1999 to 2021. Branded antibiotic usage and spending (left chart) show a steady decline, with usage decreasing from 15 billion to 9 billion standard units (SUs) and spending dropping from $19 billion to $8 billion. In contrast, generic antibiotic usage and spending (right chart) have surged. Usage rose from 25 billion to 63 billion SUs, while spending increased modestly from $5 billion to $26 billion. The data highlight a shift towards generics due to lower costs, diminishing returns for branded antibiotics, and the economic pressures affecting antibiotic innovation.

Thirdly, a new antibiotic entering the market faces significant competition from existing products. The presence of multiple analogues saturates the market, reducing the market share for new entrants. For instance, delafloxacin (Baxdela®)- a fluoroquinolone antibiotic developed by Melinta Therapeutics to target the WHO high-group pathogen Methicillin-Resistant *Staphylococcus aureus* (MRSA)- generated only US$11 million in sales during its first 12 months on the market, as the pre-existing competitor linezolid was already widely available and in use. Similarly, meropenem-vaborbactam (Vabomere®), developed for carbapenem-resistant Gram-negative infections, earned just US$11 million in its first year due to strong competition from the existing ceftazidime-avibactam (Avycaz®)^59^. Moreover, it is estimated that a new antibiotic must generate a minimum revenue of US$300 million annually to be commercially sustainable and profitable. However, the entire market for antibiotics targeting carbapenem-resistant Enterobacteriaceae (CRE)- a group of critical, life-threatening pathogens- is valued at only US$289 million per year^60^. This highlights the harsh economic reality that, despite urgent need, the market is too narrow for companies to achieve a return on investment on more than one antibiotic within a given niche^58^. Furthermore, branded antibiotics have to combat their cheap, generic counterparts. Since the past two decades, a global rise in the use of generics has been observed, whereas the on-patent antibiotic market has fallen from US$19 billion in 2001 to US$8 billion in 2021^61^ (Fig. 6).

Fourthly, the models for reimbursing other therapeutic drugs are usually sales-based, meaning that pharmaceutical companies receive a return on investment proportional to the sale of the drug, and the greater the sales volume, the greater the return. This model, however, does not apply to the antibiotic market due to antimicrobial stewardship concerns. Since predicting the emergence of resistance to existing antibiotics is difficult, new antibiotics must remain available as backup options but have to be kept on the ‘top shelf’ for reserved use^21^. Thus, there is no single business model that achieves both profit and conserved use for antibiotics simultaneously, necessitating the development of new, sustainable ones that could ensure profit for pharmaceutical companies while allowing for rational antibiotic use. Furthermore, hospitals utilise a bundled payment method, called Diagnosis-Related Group (DRG), for their reimbursement, where a fixed amount covers the medical procedure as well as the price of the drugs used. If newer, more expensive antibiotics are used, it would cover a rather large portion of the reimbursement amount and disturb the hospital budget^62^.

**Fig. 7:**
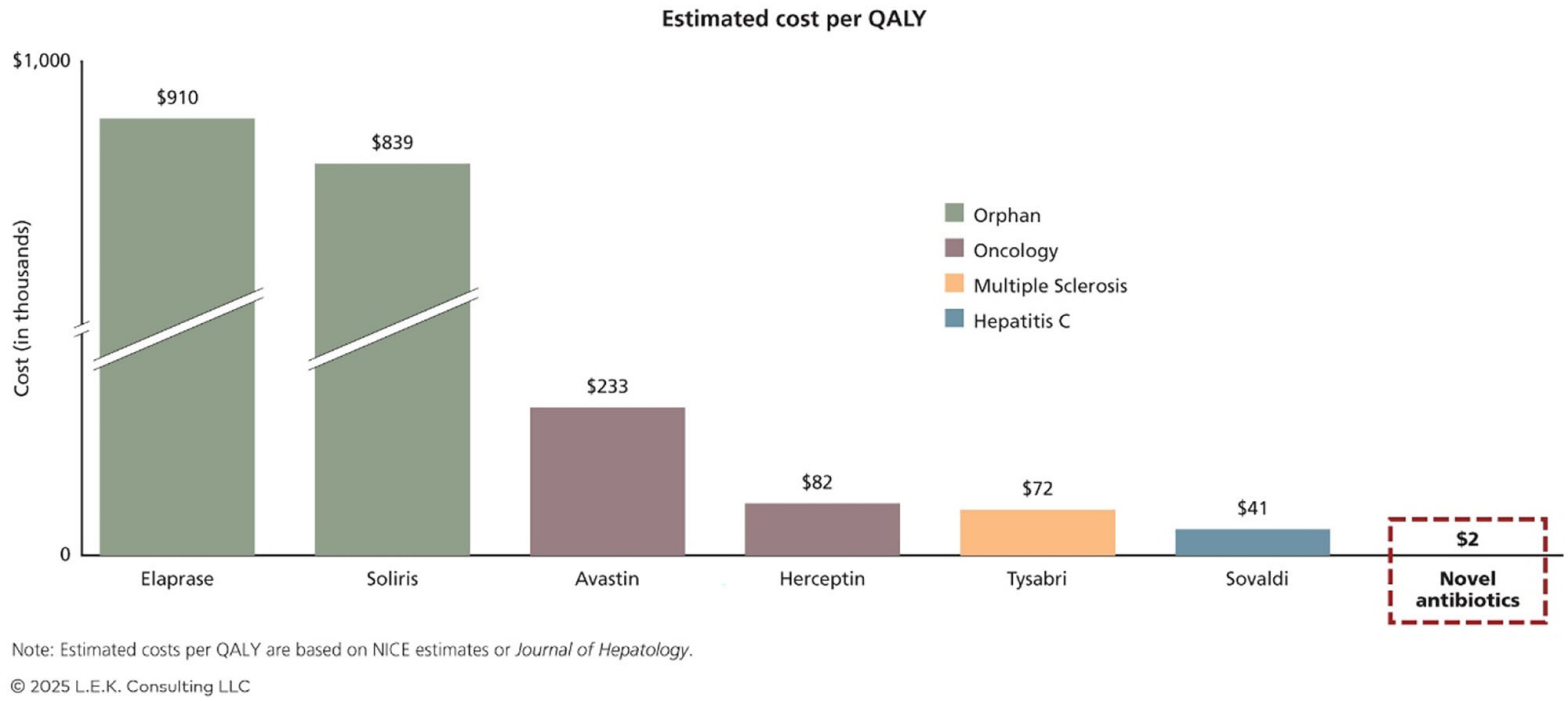
Comparison of the QALY values of different pharmaceutical therapeutics and antibiotics (Adapted from The Paradox of Antibiotics Pricing, L.E.K Consulting, with permission). The figure compares the estimated cost per quality-adjusted life year (QALY) for various drugs across therapeutic areas. Orphan drugs like Elaprase and Soliris have the highest costs per QALY, at $910,000 and $839,000, respectively. Oncology drugs such as Avastin ($233,000) and Herceptin ($82,000) demonstrate relatively lower but significant costs. Tysabri, used for multiple sclerosis, costs $72,000 per QALY, while the hepatitis C drug Sovaldi costs $41,000. In stark contrast, novel antibiotics are valued at just $2000 per QALY, as highlighted in a red box. This comparison emphasises the undervaluation of antibiotics despite their critical role, which reflects the financial and pricing challenges that disincentivise investment in antibiotic development.

Finally, antibiotic prices are kept lower than those of new anti-cancer or autoimmune drugs, as they need to be accessible to the wider population. The paradox is that even though novel antibiotics are urgently needed, they are highly undervalued. Quality-adjusted life years (QALY) are a tool that measures the cost-effectiveness of pharmaceuticals and helps companies assess their value. While the QALY values for orphan drugs (drugs used to treat rare diseases) or anti-cancer agents are significantly high, around US$900, it is as low as US$2 for antibiotics^63^ (Fig. 7). While such cost-effectiveness is beneficial for society in ensuring accessibility, it is a major blow to the pharmaceutical companies that spend USD billions developing them.

Given the complexities and market struggles associated with antibiotic development, several major pharmaceutical players have left the field. Current research and development in antibacterials is pioneered by academia and small and medium enterprises (SMEs), but these entities have also suffered from disastrous losses recently. In 2020, the US biotech company Tetraphase, which had previously brought the antibiotic drug eravacycline (Xerava®) to market, was sold due to substantial losses. Despite over a decade of effort of bringing the drug to market and achieving FDA approval in 2018, the company faced significant challenges^64^. After reaching a peak market capitalisation of US$1.8 billion, Tetraphase experienced declining sales by late 2019; within 5 years, the company’s value became worthless^65^. Another biotech firm, Achaogen, filed for bankruptcy recently. Achaogen was the developer of plazomicin (Zemdri®), an aminoglycoside developed for use in urinary tract infections caused by CRE. After being approved for use by the FDA in 2018, the antibiotic generated sales of less than US$1 million and ultimately filed for bankruptcy a year later in 2019^66^. The fact that both of these companies successfully developed and received regulatory approval for new antibiotics but still went bankrupt, with poor revenue from the approved antibiotics playing a key role, shows how broken the antibacterial market is.

Realising the severity of the antibiotic crisis, several organisations have started offering funding for promoting antibiotic R&D. However, this funding is sufficient to sustain only the preclinical phase of development. The actual cost of developing drugs rises in the later clinical and post-marketing stages, but no single investor can bear these billion-dollar costs completely^67^. Having public-private partnerships and other global platforms is hence essential to ensure that antibiotic R&D makes the transition from benchside to bedside.

## Regulatory challenges

The economic hurdles of antibiotic development are complemented by regulatory challenges, which many blame for the current pipeline scenario. In 2006, the antibiotic telithromycin was found to cause rare but life-threatening hepatotoxicity only after its approval by the regulatory agencies. Similarly, the fluoroquinolone antibiotic trovafloxacin was withdrawn from the market just three years after approval, due to a high incidence of drug-induced liver injury. To prevent the recurrence of such incidents during antibiotic approval, regulatory agencies have introduced more stringent guidelines over the past decade^68,69^. Although these guidelines aim to prioritise the safety profile of antibiotics, they have complicated the process of gaining marketing approval. Non-inferiority clinical trials, i.e., trials demonstrating that the new drug is not inferior to the standard comparator, are required by the regulatory agencies. However, these trials need to have a low non-inferiority margin (less than 10%), which is challenging to design and interpret. Such trials also require a larger sample size and involve greater costs^68^. Furthermore, when several non-inferiority trials are conducted, the actual effectiveness of the drugs may reduce over time^70^. The stringent requirement of a narrow non-inferiority margin led to the rejection of iclaprim, a diaminopyrimidine antibiotic, intended for use against multidrug-resistant Gram-positive pathogens. According to the regulatory agencies, phase 3 clinical trials of the drug did not prove its efficacy as its non-inferiority margin was beyond accepted limits at −11.7%, falling outside the accepted margin. Regulatory approval was again sought years later, but the application was once again refused due to additional toxicity testing requirements. After years of struggling to gain FDA approval, the development of iclaprim was ultimately halted^71,72^. Had iclaprim been approved, it could have served as a valuable antibiotic for treating serious skin infections and hospital-acquired bacterial pneumonia.

The case of iclaprim is not a unique one; the regulatory framework surrounding antibiotic development and approval has created problems for many new antimicrobials. In 2006, an application for the new antibiotic faropenem medoxomil was submitted to the FDA for four clinical indications. It was supported by the successful results of 11 clinical trials as well as a safety database, yet the FDA did not approve the drug; instead, additional clinical data was requested. Furthermore, the authority also demanded superiority studies for the approval of two indications. While the usual requirement has been the demonstration of antibiotic efficacy through non-inferiority trials, a sudden shift to the requirement of superiority trials greatly challenged faropenem’s development. The developers, Daiichi Asubio Pharma, also stated that an additional two years would be required to undertake further studies, which would involve greater costs and manpower^73^.

Regulatory uncertainty proved to be a large obstacle for Paratek Pharmaceuticals as well. Paratek, a Boston-based company, was developing a new tetracycline for the treatment of skin infections and bacterial pneumonia. It managed to reach phase 3 clinical trials, but with the uncertainty of FDA guidelines surrounding the design of the clinical trial and the trial endpoints, the company had to suspend the antibiotic development for around four years, resulting in a significant economic loss. Fortunately, the FDA addressed the uncertainty eventually, and with the introduction of the GAIN Act (an Act passed to stimulate new antibiotic R&D), the company was able to launch the product into the market. Although Paratek was able to survive these challenges, other small companies launched around the same time struggled, and had to ultimately abandon their antibiotic development^74^.

The design of clinical trials for antibacterials is challenging since specific pathogen-caused infections have to be considered. Given that AMR is widespread and caused by multiple different bacteria, identifying and recruiting patients with such pathogen-specific infections is difficult, as they are fewer in number^75^. This challenge was recently faced by the new aminoglycoside antibiotic plazomicin. In a clinical trial that enroled patients with urinary tract infections, plazomicin showed excellent results when compared with the standard available treatment. However, when a trial was undertaken to study the drug in carbapenem-resistant infections, only 39 patients could be recruited out of 2000 screened^76^. This exemplifies the struggles of conducting clinical trials; while new antibiotics are urgently needed for serious infections, the enrolment of patients with these infections is almost nil.

The inclusion criteria for patient enrolment are also complex. According to new regulations, existing antibiotics cannot be administered to patients before the trial; on the other hand, patients recruited with chronic infections are required to be given multiple intravenous doses of antibiotics. The former excludes the involvement of critically ill patients, while the latter of non-critically ill patients, overall complicating the trial requirements^43^. Furthermore, to minimise errors in determining the efficacy of the test drug, ventilator-associated pneumonia trials are required to recruit subjects who have not received any prior treatment. However, it is largely unethical to withhold treatment from patients infected with these high-mortality-associated diseases. As a result, the recruitment of patients in such trials is low, and generalising the trial findings becomes difficult^77^.

The varying regulatory requirements among the global authorities also hinder the antibiotic approval process. Currently, regulatory requirements are not fully harmonised across different geographical territories, and differences still exist in areas such as clinical trial protocols, patient recruitment strategies, and market approval processes. Although several agencies have worked to align the regulatory framework, further steps still need to be taken to facilitate a streamlined approval process^78^. The conflict between adaptations of the regulatory system to aid antibiotic approval and the point where further adjustments cannot be made continues even today, but remains inconclusive among the authorities themselves. Furthermore, even if regulatory authorities compromise to some extent to allow marketing authorisation with limited clinical data, the health technology assessment (HTA) agencies are firm on the requirement of substantially detailed information. The failure to provide this additional data could potentially hinder economic reimbursement and deter the company from marketing the drug. What further accelerates the problem is the dilemma of whether approving antibiotics with limited data would be risky (because safety issues could go unnoticed) or a potential advantage (because an infection with limited treatment options could benefit from a new drug that could perhaps restrict the spread of resistance)^79^.

Clinical trials and regulatory approval are critical steps in the development of any new drug, but they are also the most time and capital-consuming. While proving the safety and efficacy of new drugs is of utmost importance, these requirements should not create obstacles to the point where new drug development is severely hampered. The regulatory guidelines for antibiotic approval have been unclear and changing for a long time, discouraging pharmaceutical companies from investing in antibiotic R&D. Some efforts to streamline the approval process by the regulatory authorities around the world could significantly ease and encourage new antibiotic development.

**Fig. 8:**
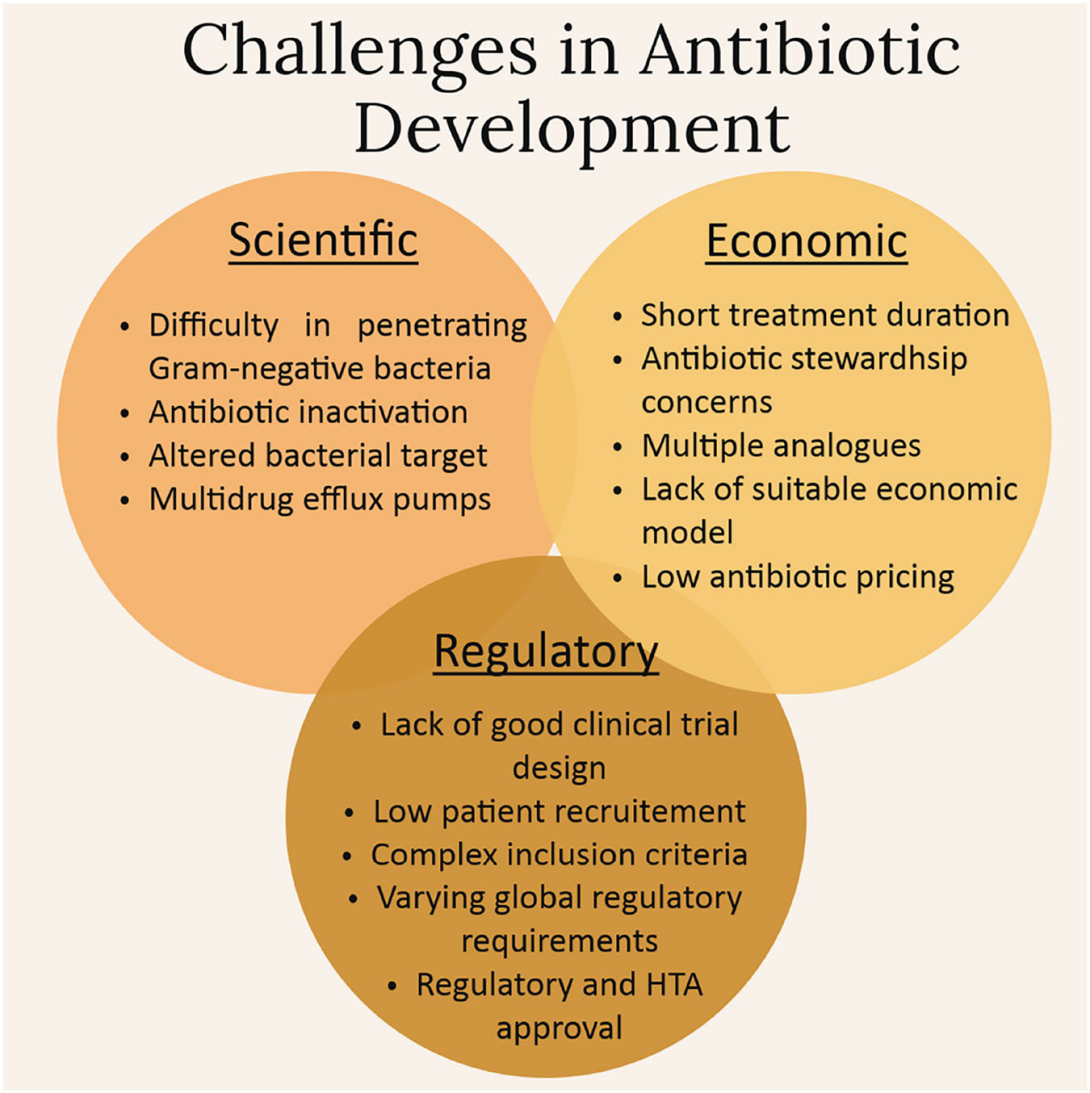
Summary of the challenges involved in antibiotic development. The figure summarises the key challenges in antibiotic development, divided into scientific, economic, and regulatory categories. Scientifically, the development of effective antibiotics faces significant hurdles such as the difficulty of penetrating the outer membrane of Gram-negative bacteria, antibiotic inactivation by bacterial enzymes, alterations in bacterial targets, and the role of multidrug efflux pumps that expel drugs from bacterial cells. Economically, antibiotics suffer from short treatment durations and restricted use due to stewardship concerns, which limit market size and profitability. The existence of multiple analogues further dilutes potential revenue, and the absence of a sustainable economic model, coupled with low antibiotic pricing, creates financial disincentives for pharmaceutical companies to invest in this space. Regulatory challenges exacerbate these issues, with clinical trials often hampered by poor design, low patient recruitment, and complex inclusion criteria. Moreover, varying global regulatory standards and difficulties in obtaining regulatory and health technology assessment (HTA) approvals further complicate the antibiotic innovation process. These overlapping barriers highlight the urgent need for a collaborative and integrated approach to revitalise antibiotic development.

In summary, the major hurdles impeding antibiotic discovery and development are three-fold: scientific, economic, and regulatory (Fig. 8). The scientific difficulties arise mainly due to bacterial physiology, and the economic and regulatory challenges due to low profitability and clinical trial complexities, respectively. It should be noted that while scientific obstacles are difficult to overcome, it is mainly the economic issues that impede antibiotic development^21^. If rational models to fund and sustain antibiotics are discovered and implemented, pharmaceutical companies and SMEs would likely rejoin antibacterial R&D and, given the broken market, find innovative opportunities to repair it.

## Revitalising the antibiotic pipeline

Whereas the antibiotic pipeline was once filled with several novel classes of antibiotics, the current pipeline is dry and in serious need of revival. Finding and implementing solutions to reinvigorate the antibiotic pipeline is of paramount importance, given that the rate of new antibiotic development is far outstripped by that of AMR emergence. A combination of approaches must be adopted to tackle this situation and ensure that AMR spread is limited. Although antibiotics have been the ‘miracle drugs’ of the 21st century, there is no doubt that the current platforms for discovering them are failing to deliver promising candidates. As financial barriers significantly hinder R&D, new business models and economic incentives must be introduced to support and promote drug discovery. Allowing a more flexible approach to the regulatory process would ensure that obstacles to new drug approval are reduced. In the following section, some solutions to revitalise the antibiotic pipeline shall be discussed.

## Solutions to scientific challenges

To rejuvenate the antibacterial pipeline, a solid foundation must be laid that enables new antibiotic discovery in the coming decades. This will likely require the integration of multiple approaches and expertise from several domains, including chemistry, microbiology, pharmacology, biotechnology, and artificial intelligence. Various scientific solutions have been proposed to address challenges in antibiotic discovery. These include the use of artificial intelligence and machine learning for library screening and hit identification^80^, physiologically-relevant and target-based screening assays^81,82^, exploration of unique microbial sources like marine environments^83–86^, endophytes^87,88^, neglected bacterial species like *Burkholderia*^89^ and other rare and unusual microbiomes and species^90–92^, advancing the understanding of permeability barriers in Gram-negative bacteria^93–95^, use of polypharmacology^96,97^ to make resistance harder to achieve, and development and use of antibiotic resistance breakers^98^. Comprehensive discussions of the solutions to scientific challenges can be found in the relevant articles.

Recently, non-antibiotic interventions have garnered much attention as potential scientific solutions to address AMR. These agents exploit the fundamental differences between bacteria (prokaryotes) and humans (eukaryotic) and provide great opportunities to expand the repertoire of antimicrobial agents. Used alongside antibiotics or alone, they can overcome the limitations of traditional antibiotic therapy, such as specificity, resistance, and efficacy. One of the most promising non-traditional antibacterial strategies is phage therapy, which uses bacteriophages to infect and kill bacteria. Phages are non-living viruses that attach to specific receptors on the bacterial surface to inject their genetic material (DNA or RNA). The phage DNA then takes control of the bacterial replication machinery to produce phage progeny, and following mass production, phage-derived proteins are activated, which lyse the bacteria^99^.

Antimicrobial peptides (AMPs) are another promising approach in this regard. These naturally occurring host defence peptides produced by all multicellular organisms, have the potential to directly kill bacteria by either disrupting bacterial membranes or targeting intracellular components and processes^100,101^. Multiple and complex mechanisms of action of AMPs impart them great efficacy while conferring activity against a range of pathogens.

Similarly, harnessing bacterial survival and dominance instincts can also be a novel strategy. Bacteria often produce toxins to inhibit or kill closely related bacterial strains to compete for resources. These toxins, called bacteriocins, allow bacteria to gain a competitive advantage over other species. Bacteriocins produced by *Pseudomonas* species, called pyocins, have shown great benefit in *P. aeruginosa* infections. They have a narrow spectrum of action and work by forming pores in the competitor’s membranes, causing intracellular leakage and ultimately death^102^. Several studies have explored the potential of pyocins as antibacterial agents and demonstrated their efficacy in targeting drug-resistant pathogenic strains, highlighting their potential as alternatives to antibiotics^103–105^. Some other non-traditional scientific approaches being developed include vaccines^106^, antibodies, nanoparticles^107,108^, and genetically engineered systems like CRISPR^109,110^, elaborate discussions on which can be found in the respective articles.

While non-traditional antibacterial strategies offer promising alternatives to conventional antibiotics, each faces unique limitations that challenge their clinical translation. Bacteriophages, for example, have a narrow spectrum of activity- beneficial for minimising off-target toxicity- but this limits their use against infections involving multiple bacterial species or strains^111^. Their use may also elicit host immune response- indirectly through antigen release during bacterial lysis- and directly via phage particle recognition^112,113^. Moreover, the lack of a well-established regulatory path for the approval impedes their therapeutic use^112^. AMPs, despite potent activity, are often unstable in vivo due to proteolytic degradation and altered activity at high physiological salt concentrations^100^. Resistance is another concern, as bacteria can employ various mechanisms to evade their activity. Among the approved AMPs, daptomycin and polymyxins have been identified to acquire resistance due to modifications in the cytoplasmic bacterial membrane, the key target of these peptides^114^. Additional barriers such as cytotoxicity, high development costs, and challenges in delivery are some other factors to be considered for their broader use. Lastly, despite the promising activity of pyocins, their narrow spectrum limited to *P. aeruginosa* strains makes them ineffective against other infections caused by other pathogens. Resistance mechanisms developed by *P. aeruginosa*, such as intrinsic immunity or mutations in proteins essential for pyocin translocation, also make them intrinsically resistant to the pyocins they produce, further reducing their effectiveness^115^. Similar to AMPs, pyocins face barriers related to proteolytic degradation, low bioavailability, high manufacturing costs, and regulatory hurdles, all of which must be addressed for their successful therapeutic development.

A more thorough consideration of the aforementioned avenues of research, including opportunities and recent developments, is beyond the scope of the present review.

## Solutions to economic challenges

Since economic problems are key contributors to the current plight of the antibiotics sector, developing and implementing strong incentives that could support and sustain the antibiotic market are of utmost importance. These incentives could encourage pharmaceutical firms and SMEs to reinvest in the antibacterial field by either supporting drug development or rewarding their efforts. Luckily, policymakers and other stakeholders have recognised the severity of financial bottlenecks in antibiotic discovery, and several types of incentives have been introduced. In the following section, existing as well as new, potential strategies shall be discussed. It should be noted that while all incentives strive to promote research and development, they are associated with specific advantages and disadvantages. Briefly, incentives can be categorised as push, pull, or mix.

## Common incentive strategies

### Push incentives

Push incentives are financial aids that pay for the research inputs of drug development. Such incentives are particularly useful for SMEs since they allow participation in new drug development by providing financial measures to translate their research from the laboratory environment to clinical settings. Examples of push incentives include tax incentives, research grants, public-private partnerships, enhancing access to research, etc.^116^.

Public-private partnerships (PPP) are collaborations that aim to share the risks and rewards of new drug development. Notable PPPs in the antibiotic domain are the Biomedical Advanced Research and Development Authority (BARDA) and New Drugs for Bad Bugs (ND4BB) in the US and EU, respectively. Following the establishment of its broad-spectrum antimicrobial programme in 2010, BARDA aims to provide late-stage clinical funding for antibiotics, as well as technical expertise to pharma companies developing novel antibacterial candidates. BARDA’s financial support has led to the successful approval of several novel antibiotics, including plazomicin, meropenem-vaborbactam, ceftazidime-avibactam, and evaracycline^117^. In 2016, BARDA also co-founded CARB-X (Combating Antibiotic-Resistant Bacteria Biopharmaceutical Accelerator), the largest antibacterial non-profit partnership, to accelerate the discovery and development of several antibacterial products. CARB-X has a vast portfolio that supports vaccine, antibiotic, and diagnostics development^118^. In Europe, the ND4BB programme was launched by the Innovative Medicine Initiative (IMI) to tackle the growing burden of AMR. It was launched in 2012 and, with a budget of EUR €700 million, plans to address all three challenges facing antibiotic development. To tackle the poor economics of antibacterials, ND4BB established the DRIVE-AB consortium, which aims to develop new commercial models that could encourage investment as well as conserved use of antibiotics^119^.

Separately, the Drugs for Neglected Diseases Initiative (DND*i*), a non-profit drug research and development organisation, and the WHO have jointly developed the Global Antibiotic Research and Development Partnership (GARDP). This initiative forms a vital component of the WHO’s action plan on antimicrobial resistance, addressing the urgent need for antibiotic therapies in LMICs, which are often overlooked by industry due to low profitability. GARDP prioritises the development of antibiotics targeting WHO-designated priority pathogens, with a strong emphasis on ensuring good antimicrobial stewardship during clinical development. Its current focus areas include antibiotics for treating sexually transmitted infections (particularly gonorrhoea) and neonatal sepsis^120^.

Grants are another push incentive where funding is provided during research to reduce economic burden and encourage researchers to participate in innovation. These research grants can be conditional, where stewardship measures are applicable for the new antibiotic, or direct. Specific grants that finance personnel training and translational research also exist^116,121^. LifeArc, a UK-based charity, is a notable example here and offers funding and support, including scientific expertise and intellectual property considerations, for the progression of early-stage antibiotic research towards clinical application^122^. Funding from venture capital groups is also available, with a prominent example being the REPAIR (Replenishing and Enabling the Pipeline for Anti-Infective Resistance) Impact Fund of Novo Holdings A/S, as commissioned by the Novo Nordisk Foundation. The fund was established in early 2018 with a budget of US$165 million and an aim to invest in early-stage therapies to combat AMR, with a focus on antibiotics targeting WHO and CDC priority pathogens. Since then, the fund has invested in a number of prominent antibiotic companies, including Entasis Therapeutics, Spero Therapeutics, and Minervax^123^.

Another key initiative is the AMR Action Fund, serving as the world’s largest venture capital fund to combat antimicrobial resistance. It has over 20 investors and seeks to raise more than US$1 billion, with a primary focus on supporting small to mid-sized biotech companies developing antimicrobial agents against priority pathogens. The fund also aims to provide expertise and scientific support to these companies, ensuring their efforts align with stewardship and sustainable antibiotic use principles^124,125^. In addition to the AMR Action Fund, the Fleming initiative presents a complementary approach, emphasising the importance of addressing AMR beyond science alone. Launched by Imperial College London and Imperial College Healthcare NHS Trust, this initiative focuses on four streams- behavioural and translational science, policy, and public engagement & involvement^126^. The initiative is opening Fleming Centre to bring together scientists, healthcare professionals, policymakers, behavioural experts, and commercial partners to co-create and deliver impactful solutions against AMR. Furthermore, the PACE (Pathways to Antimicrobial Clinical Efficacy) initiative aims to address the financial barriers faced by academia and SME. It has been jointly developed by UK research organisations LifeArc, Medicines Discovery Catapult, and Innovate UK^127^. As a £30 million initiative, PACE will prioritise new medicines and diagnostics, develop their target profiles, and fund projects that address the priority pathogens. It aims to establish a more efficient ecosystem in the UK for AMR research and development and increase confidence in tackling AMR.

Although these initiatives are a great effort to stimulate antibacterial R&D, it is uncertain whether the magnitude of funding they provide is sufficient to match the need of the situation. To properly reinvigorate and sustain the antibiotic pipeline, additional push funding, between $250 and $400 million globally per year, is required, along with pull incentives, as per a European Commission study^128^. Additionally, funding must support the entire drug development process rather than a single stage. Adequate financial resources are essential during the early stages to promote innovation and generate novel candidates, while investment is equally critical in the later stages, as clinical development entails significant costs.

It is also important to recognise that funding alone cannot compensate for the expertise gap and shortcomings that the SMEs and academia face during drug discovery. Drug discovery is an extremely complex process, particularly in the early stages, where identifying candidates with both significant societal impact and strong potential requires substantial technical expertise. Despite these incentives, early stages- particularly lead characterisation and optimisation- currently remain largely underfunded, resulting in critical funding gaps^129^. Similarly, tax incentives from the government reduce the tax burden and encourage new molecule R&D. Another example of a push mechanism is open access to research, which, by allowing research in the public domain, fosters collaboration and knowledge sharing, while minimising effort duplication^130^.

Although push incentives are advantageous in reducing the initial costs of research and encouraging development, they are associated with a high risk of project failure, precluding their implementation. Moreover, research grants do not guarantee the project’s success, and it is often believed that scientists with funding are not committed to research. Since drug developers have more knowledge of their projects than the funders, there is a possibility that this situation can be taken advantage of to secure funding^130^. Nonetheless, push incentives are a common strategy to promote antibiotic R&D and may be complemented by other incentives for optimal benefit.

### Pull incentives

Pull incentives are financial measures that pay for the research and development outputs by providing a return on investment. In other words, while push incentives ‘encourage’ drug development, pull incentives ‘reward’ it. Multiple pull incentives exist, such as market entry rewards (partially delinked or fully delinked), higher reimbursement, diagnosis-related group carveout, patent exclusivity, tradeable exclusivity vouchers, etc.

Market entry rewards (MER) are a strategy whereby the innovation is funded by a series of payments based on the units of antibiotics sold. However, as antibiotic stewardship concerns limit the application of this model, variations such as partially or fully delinked MER have been introduced. In partially delinked market entry rewards, smaller payments are provided annually in addition to the usual unit sales-based revenue^131^. This ensures profit even with a low sales volume of drugs, as is desired with antibiotics. Several methods for implementing MER have been proposed, such as fund allocation only to antibiotics targeting priority pathogens, case-dependent allocation, stewardship-based MER models, etc.^132^. In a fully delinked MER model, sales volume does not drive the funding. Incentive payments solely fund the antibiotics and are subject to stewardship conditions, i.e., the company must ensure restricted sale of antibiotics to receive the payments^131^.

An important step towards partly delinking incentives from antibiotic sales has been taken by the UK in 2022 through the Antimicrobial Product Subscription Model. Under this model, the National Health Service (NHS) will pay to pharmaceutical companies an annual subscription fee to procure and use new antibiotics^133,134^. Based on the NICE’s (National Institute for Health and Care Excellence) assessment of the eligibility and value of the medicine, it will be placed in one of four bands, receiving subscription fees between £5 million and £20 million per year. Thus far, two subscriptions have been awarded under the pilot model to Pfizer and Shionogi for ceftazidime-avibactam and cefiderocol, respectively, for use against drug-resistant Gram-negative bacteria^135^. The USA also aims to undertake a similar approach to foster the discovery and development of new antibiotics through the PASTEUR (Pioneering Antimicrobial Subscriptions to End Upsurging Resistance) Act. Introduced in the US Senate and House of Representatives as legislation in 2021, PASTEUR would pay antibiotic innovators a fixed annual contract amount based on the novelty, efficacy, and public health value of the antibiotic^136^. These incentives would last 5 years to the medicine’s patent life, with conditions requiring antibiotic stewardship and global access to these novel therapies.

A similar revenue guarantee pull model has been proposed by the EU, where the European Medicines Agency (EMA) will assess a novel antimicrobial agent for revenue guarantees. EU Member States will then evaluate the agent and accordingly decide their participation. At the end of the contract, Member States will pay the difference between the financial commitment and actual units consumed to the innovator companies. This system ensures that companies are fairly compensated and encourages the Member States to use the antibiotic responsibly, to avoid unnecessary costs^137^.

Both- the subscription and revenue guarantee model- are volume-delinked pull models that compensate innovators based on their innovation and efforts, rather than on unit sales, ensuring they are fairly rewarded while adhering to antimicrobial stewardship practices. They rightly address the main concerns in antibacterial development- the pressing need for novel therapies and low antibiotic sales due to stewardship measures. However, their effectiveness is limited without global coordination. Since it is mainly the LMICs with high antibiotic consumption and misuse, implementing such models in high-income countries alone is insufficient to tackle AMR globally. Additionally, whether mere funding will attract companies to develop novel, innovative antibacterials is also worth considering. Developing a new antibacterial compound from scratch comes with a substantially higher risk, cost, and effort, as opposed to analogue development, and companies may be hesitant to pursue such risky ventures, even with incentives. Moreover, as the effectiveness of an antibacterial agent can only be measured after its prolonged use, it is unclear how the UK’s subscription model will assign a ‘value’ to new antibiotics. There are also concerns that these models may result in counterproductive consequences, such as companies withholding innovation to capitalise on higher rewards as AMR intensifies and strategic lobbying to maximise profits^134,138^. Finally, whether antibiotics developed through these models will be accessible and affordable to LMICs is currently unknown^128^. Reimbursing antibiotics is another push mechanism whereby the drug would be sold at a higher price. This would ensure that the use of the drug is conserved and under stewardship measures. However, this is not possible in hospitals since the Diagnosis-Related Group (DRG) system is generally employed for reimbursement, which depends on the medical procedure and not the individual item. As a solution to this, independent reimbursement of antibiotics has been suggested through the DRG carve-out model, by which hospitals could be reimbursed at a higher value for new antibiotics^139^.

Another innovative pull incentive is the tradeable exclusivity voucher. This voucher is awarded to the innovator companies that meet the set criteria and allow them to extend the patent period of their product for a considerable time or sell it to other companies. By extending the exclusivity period, the innovators can receive a significantly higher return on investment, as there would be fewer generics to compete against, and by selling it to other companies, they can receive instant gain. In either case, the innovators could benefit. Tradeable exclusivity vouchers have the potential to be a great incentive for stimulating antibiotic R&D as they would provide a means to improve the profitability of marketed antibiotic product^139^.

All pull incentives, unfortunately, have some disadvantages. While MERs are one of the most promising mechanisms for funding antibiotics, sustaining fully delinked MERs is difficult, as the funding would rely solely on payments that could total up to a billion per antibiotic^50^. Strategies like high reimbursement of antibiotics, while ensuring conserved use, reduce access to the general population. A similar issue occurs with tradeable exclusivity vouchers, where exclusivity extensions limit generic drugs in the market and reduce their availability to patients. Lastly, DRG carve-out and partially delinked MERs are linked with sales volume, hence, profitability will always be at the expense of antimicrobial stewardship.

### Mixed incentives and other models

As the name suggests, mixed models involve a combination of both push and pull incentives or the combination of several incentives in a single model. Given the limitations of both mechanisms, combining them in a mixed model can help achieve their maximum potential. The implementation of a mixed model has been strongly suggested previously as a key incentive for promoting antibiotic discovery and development^132^.

One such example of a mixed model is the Options Market Award model, where options would be available to a third-party payer to purchase a specific volume of the antibiotic being developed. If the drug is purchased during the early stages of development, the cost would be low, but there would be a risk of the product not making it to the market. If purchased at a later stage, the cost would be significantly higher. The third-party payer would benefit in either case, as they fund the development of the desired product if purchased early, and receive it at a much-discounted rate compared to the market cost if purchased later^140^.

The mixed model could also include policies like the Orphan Drug Act that was implemented in the USA to promote R&D for orphan drugs. The Act provides exclusive marketing rights to the product innovators who develops these drugs for several years. In addition, other advantages are bestowed, such as regulatory fee wave-off, tax credit provision, and regulatory priority review for the product. The implementation of this Act saw a significant increase in research and clinical trials for orphan drugs^141^. Perhaps, such models with a combination of multiple incentives could benefit antibiotic research as well, where each model could help overcome the limitations of others.

Other mechanisms where antibiotic use is taxed could be employed. However, as it would enhance the prices and negatively affect accessibility, such an incentive would likely only be useful in non-human antibiotic use-settings such as agriculture. These taxes would not only promote stewardship but also allow the generated funds to be used for innovation purposes^142^. Antibiotic taxing could also refer to an investment charge, where pharmaceutical companies are made to invest a certain amount in antibacterial R&D. Because antibiotics are essential for almost every medical procedure, the entire pharmaceutical industry should contribute to developing these medicines^50^.

## Other potential strategies to stimulate antibiotic R&D

Despite several push, pull, and mixed mechanisms for promoting investment, the antibiotic development field still lacks a solid model that overcomes all the economic limitations. The model, while efficiently supporting the development of new antibiotics, should be feasible and viable in the long term. It should be coordinated worldwide to ensure commensurate access to this important class of medicine.

Singer et al. propose a two-pronged approach consisting of short-term and long-term goals to ensure a steady and sustainable flow of antibiotics. According to their approach, the existing push-pull models should be strengthened in the short term, as they promote the already available knowledge, skill, and staff- and abandoning them abruptly could result in the loss of decades of commercial expertise. For the long-term goal, however, they suggest the establishment of a globally coordinated public-funded institute for antibiotic R&D. Since antibiotics are a universal essential commodity, the participating countries could form a pool of funding to sustain this institute. All research carried out here would be open, meaning the research and development and financial burden would be shared. Such an institute would fund the scientists to undertake antibacterial R&D and would foster collaboration between them, along with knowledge sharing. It would also prevent research duplication and ensure a viable platform that delivers antibiotics according to the need^143^.

Another strategy is implementing three councils that have distinct functions yet work collaboratively to incentivise R&D, promote stewardship, and develop antibiotics as per medical needs. According to this idea, one of the councils would support the existing PPPs and grants to stimulate antibiotic innovation. The second council would manage the funding reward that all participating nations would contribute to. All drug innovators would subscribe to this funding, which would be reimbursed to cover the costs of initial R&D for the first five years and the cost of production after that. The third council would be in charge of distributing the developed drugs according to their medical need, and also for communicating the need for desired antibiotics to the first two councils^144^.

To overcome the limitations of the traditional push mechanisms, Morel et al. suggest another feature to be added to the MER model called the antibiotic susceptibility bonus (ASB). ASB would be the performance-dependent component of MER that could be allocated, provided the innovator antibiotic meets the specified susceptibility targets. Given that resistance spreads with increased use of antibiotics, such a model would encourage the product developers to promote the antibiotic with conservation by financially rewarding it. Moreover, it would enhance the sustainability of the MER model by ensuring that only products with appropriate activity profiles receive funding^145^.

**Fig. 9:**
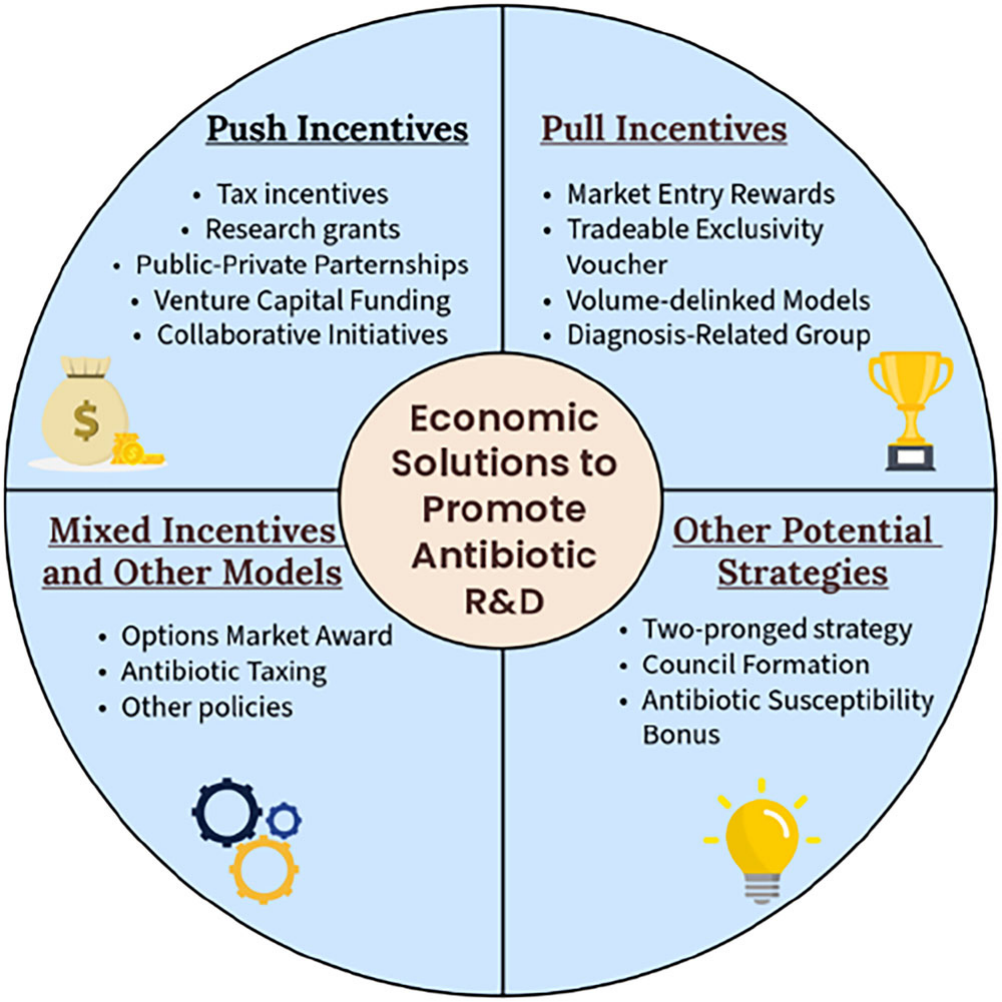
Different types of incentive strategies as a solution to promote antibiotic R&D. The figure highlights economic strategies to promote antibiotic R&D, categorised into four domains. Push incentives include tax benefits, research grants, public-private partnerships, venture capital funding, and collaborative initiatives targeting early-stage R&D support. Pull incentives focus on market entry rewards, tradeable exclusivity vouchers, volume-delinked payment models, and diagnosis-related groups, offering financial returns post-market approval. Mixed incentives and other models combine approaches like options market awards, antibiotic taxing, and tailored policies to bridge funding gaps. Other potential strategies suggest a two-pronged approach, council formation for antibiotic oversight, and an antibiotic susceptibility bonus. These measures collectively aim to address the financial challenges limiting antibiotic innovation and facilitate sustainable R&D investments.

As the pharmaceutical industry is the largest single source of relevant skill, experience, manufacturing, and capital with regard to antibiotic R&D, it is extremely necessary that these companies rejoin the field. Economic incentives (Fig. 9) appear promising in this regard, as they can potentially motivate innovators to drive efforts in developing drug candidates by rewarding them. Implementing efficient business models that sustain antibiotic discovery yet ensure their rational use would solve the majority of challenges that antibacterial R&D currently faces.

## Solutions to regulatory challenges

Several regulatory pathways have been introduced to support the development and exploration of investigational antimicrobial agents, especially those targeting resistant infections. These include Breakthrough Therapy Designation (FDA), Fast Track Designation as well as Priority Review (FDA and EMA), Conditional Marketing Authorisation (EMA and MHRA), and the Priority Medicines (PRIME) scheme (EMA). Although some of these pathways are not exclusive to antimicrobial agents, they can facilitate the development of new antibiotics.

The regulatory aspects of antibiotic R&D have been more positive for the past few years as authorities have recognised the importance of having a more streamlined approval process. For instance, the guidelines for conducting trials for skin infections and hospital and ventilator-acquired bacterial pneumonia were updated in 2014 by the US FDA and now allow wider inclusion criteria, a larger non-inferiority margin, and the enrolment of patients who have received antibiotic therapy before 24 hours of enrolment. The endpoints for these studies were also reconsidered and are now based on overall survival and not on disease-related complications, making the trial analysis simpler for pharmaceutical companies^146^.

In Europe, the COMBACTE project was established as a part of the ND4BB programme to ensure the feasibility of clinical trials. Initiated in 2013, it aims to build a pan-European clinical trial and research network as well as create robust, sustainable frameworks for the scientific evaluation of new antibiotics. Such projects allow for the efficient recruitment of patients for clinical trials, which is often a barrier for trials focusing on multidrug-resistant infections. They also ensure that the trial design is consistent and standardised, enhancing the reliability of the results. With three main components- CLIN-Net, LAB-Net and STAT-Net, COMBACTE provides a unified platform to advance antimicrobial research and development across Europe^119^.

Considering the urgent need for treating severe bacterial infections but the lengthy and often time-consuming clinical trials, the Limited Population Antibacterial Drug (LPAD) pathway was introduced by the US FDA under which antibiotics to treat infections with an unmet clinical need receive expedited approval This pathway allows the drugs to be studied in shorter, more rapid clinical trials with a limited number of subjects. It reflects an effort to incentivise the development of critical antibiotics while ensuring safe and effective use in vulnerable populations. The approved drugs also follow specific labelling indicating their limited safety data, to prevent their use by the general population^146^.

In addition to LPAD, drugs can be designated with a Qualified Infectious Disease Pathogen (QIDP) status by the FDA if they are used to treat life-threatening infections caused by resistant pathogens. The QIPD status would allow the extension of the drug’s market exclusivity, and provide a fast-track designation and priority review, to ensure its rapid approval and entry into the market^147^.

The adjusted requirement of the clinical trial designs and the establishment of pathways to expedite drug approval reflect the approaches undertaken by the regulatory agencies to facilitate marketing approval of new antibiotics. These changes are expected to reduce the associated regulatory burden and support the development of new antibacterials.

The regulatory solutions discussed here do not question the need for patient safety or rigorous standards. Instead, they highlight the need for adjusted guidelines tailored to antibiotics, which face distinct scientific and economic barriers. Antibiotics are short-course treatments used for serious infections and are subject to stewardship. They do not generate high-volume sales, making lengthy and costly trials difficult to sustain. There is no need to reduce safety standards or adopt emergency-style fast-tracking as seen with COVID-19 vaccines. Antibiotics are well-characterised small molecules with an established development process. However, some trial requirements, such as very narrow non-inferiority margins, strict inclusion criteria, and large recruitment targets, should be reconsidered. Greater flexibility in trial design, acceptance of real-world data, and alignment between regulatory agencies could support approval without compromising safety. A transparent, science-based approach to regulatory adaptation, clearly communicating the rationale behind modified trial designs or data requirements, can maintain public trust while expediting the development of urgently needed antibiotics.

## Conclusion and future perspective

Antibiotics are the pillars of modern medicine as their use is a crucial aspect of almost all medical procedures. In this regard, the rapid emergence and spread of antimicrobial resistance is an urgent global problem. Patients are dying, pan-resistant infections are being reported more frequently, and the world economy is increasingly feeling the cost of the crisis. At the same time, the development of new antibiotics has stagnated over the past couple of decades, and the pipeline of new therapies has become severely limited. Disregarding the development of analogues of existing antibiotic scaffolds, only a few therapies have attained market approval since the year 2000. Collaborative efforts by academia, industry, world governments, charities, policymakers, and regulatory authorities have to be undertaken to remedy this complex situation.

While several new antibiotics are in the preclinical and clinical stages, the development process is extremely cumbersome with scientific, economic, and regulatory challenges surrounding it. Regarding the economic challenges, new antibiotics have limited sales owing to the short antibacterial treatment courses and stewardship concerns. Incentivising antibiotic discovery and creating new business models that sustain these developed antibiotics is hence extremely crucial. Push incentives such as grant and venture capital funding, and pull mechanisms such as market entry rewards should be implemented to encourage drug developers to invest in the field. A mixed model that involves both types of incentive could also prove useful. An open research platform that allows knowledge sharing and fosters collaboration between scientists can accelerate the drug development process further. Regarding the regulatory challenges, the regulatory framework surrounding antibiotic development needs to be streamlined and coordinated globally to harmonise the marketing approval process. Regulatory guidelines, clinical trial endpoints, and trial design specifications should be well-defined and communicated clearly to ensure that clinical trials are simplified while still evaluating safety and efficacy.

While the situation appears bleak, there is still cause for hope. Public awareness of the AMR crisis has risen over the past 10 years, and public-private partnerships, venture capital funds, and new initiatives are supporting antibiotic development at various stages by not only providing funding but also technical expertise and commercialisation support. The WHO and member countries are actively taking efforts to tackle AMR and foster the development of novel antibacterials. Efforts to make the regulatory framework more supportive have begun, as seen with the introduction of the LPAD pathway and QIDP status. The number of candidates in the pipeline is also encouraging, with 7 new antibiotics being approved since 2016 and multiple others in various stages of clinical trials^148^. Overall, inputs from stakeholders of different sectors have started shaping a new path for the antibiotic field, which has long faced significant challenges. Time will tell if the myriad scientific, economic and regulatory solutions being implemented can turn the battle against AMR in humanity’s favour.

## Data Availability

No datasets were generated or analysed during the current study.
